# One-year surveillance of SARS-CoV-2 transmission of the ELISA cohort: A model for population-based monitoring of infection risk

**DOI:** 10.1126/sciadv.abm5016

**Published:** 2022-04-15

**Authors:** Christine Klein, Max Borsche, Alexander Balck, Bandik Föh, Johann Rahmöller, Elke Peters, Jan Knickmann, Miranda Lane, Eva-Juliane Vollstedt, Susanne A. Elsner, Nadja Käding, Susanne Hauswaldt, Tanja Lange, Jennifer E. Hundt, Selina Lehrian, Julia Giese, Alexander Mischnik, Stefan Niemann, Florian Maurer, Susanne Homolka, Laura Paulowski, Jan Kramer, Christoph Twesten, Christian Sina, Gabriele Gillessen-Kaesbach, Hauke Busch, Marc Ehlers, Stefan Taube, Jan Rupp, Alexander Katalinic

**Affiliations:** 1 Institute of Neurogenetics, University of Lübeck and University Hospital Schleswig-Holstein, Campus Lübeck, Lübeck, Germany.; 2 Department of Neurology, University of Lübeckand and University Hospital Schleswig-Holstein, Campus Lübeck, Lübeck, Germany.; 3 Institute of Nutritional Medicine, University of Lübeck, Lübeck, Germany.; 4 Department of Medicine I, University Hospital Schleswig-Holstein, Lübeck, Germany.; 5 Department of Anesthesiology and Intensive Care, University Hospital Schleswig-Holstein, Lübeck, Germany.; 6 Institute of Social Medicine and Epidemiology, University of Lübeck, Lübeck, Germany.; 7 Institute of Virology and Cell Biology, University of Lübeck, Lübeck, Germany.; 8 Department of Infectious Diseases and Microbiology, University of Lübeck and University Hospital Schleswig-Holstein, Campus Lübeck, Lübeck, Germany.; 9 Department of Rheumatology and Clinical Immunology, University of Lübeck and University Hospital Schleswig-Holstein, Campus Lübeck, Lübeck, Germany.; 10 Lübeck Institute of Experimental Dermatology (LIED), University of Lübeck, Lübeck, Germany.; 11 Health Protection Authority, Lübeck, Germany.; 12 Research Center Borstel, Leibniz Lung Center, Borstel, Germany.; 13 German Center for Infection Research (DZIF), Partner site Hamburg-Lübeck-Borstel-Riems, Borstel, Germany.; 14 LADR Laboratory Group Dr. Kramer & Colleagues, Geesthacht, Germany.; 15 Perfood GmbH, Lübeck, Germany.; 16 University of Lübeck, Lübeck, Germany.

## Abstract

With newly rising coronavirus disease 2019 (COVID-19) cases, important data gaps remain on (i) long-term dynamics of severe acute respiratory syndrome coronavirus 2 (SARS-CoV-2) infection rates in fixed cohorts (ii) identification of risk factors, and (iii) establishment of effective surveillance strategies. By polymerase chain reaction and antibody testing of 1% of the local population and >90,000 app-based datasets, the present study surveilled a catchment area of 300,000 inhabitants from March 2020 to February 2021. Cohort (56% female; mean age, 45.6 years) retention was 75 to 98%. Increased risk for seropositivity was detected in several high-exposure groups, especially nurses. Unreported infections dropped from 92 to 29% during the study. “Contact to COVID-19–affected” was the strongest risk factor, whereas public transportation, having children in school, or tourism did not affect infection rates. With the first SARS-CoV-2 cohort study, we provide a transferable model for effective surveillance, enabling monitoring of reinfection rates and increased preparedness for future pandemics.

## Teaser

Cohort surveillance of SARS-CoV-2 transmission effectively monitors infection rates and identifies and debunks risk factors.

## INTRODUCTION

The coronavirus disease 2019 (COVID-19) pandemic has affected >360 million confirmed cases and >5.6 million deceased patients worldwide [(*1*); www.coronatracker.com, accessed 27 January 2022). Although research activities on all aspects of severe acute respiratory syndrome coronavirus 2 (SARS-CoV-2) infection and COVID-19 are unprecedented in speed and scope, information and strategies currently remain scarce on (i) long-term development and dynamics of incidence and prevalence of SARS-CoV-2 infection and reporting in fixed cohorts, (ii) identification of risk factors for conversion from low- to high-prevalence areas, and (iii) establishment of effective, population-based surveillance strategies.

Population-based studies, such as the one undertaken in the Icelandic population at the beginning of the pandemic, are typically cross-sectional or cover short time intervals (*2*). Likewise, antibody prevalence has been assessed cross-sectionally or spanned short time periods in specific regions (*3*–*8*), and only select subgroups have been followed longitudinally (*9*, *10*). Of further note, effects of containment measures are rarely assessed directly but rather modeled (*11*–*15*), frequently based on reported infections (*16*), and unlikely to be widely generalizable from one geographic and/or cultural setting to another.

### Our contribution

The present study is based on ~20,000 virus polymerase chain reaction (PCR) and anti–SARS-CoV-2 spike protein (S1) immunoglobulin G (IgG) antibody tests, as well as >90,000 detailed app-based datasets on symptoms, mobility, pandemic-related behavior, and quality of life. Thus, we here report the first longitudinal, prospective cohort study surveilling ~3000 people who represent ~1% of the population living in our catchment area, a major tourist region on the Baltic Sea in Northern Germany for the entire first year of the pandemic. Cohort follow-up spans a highly dynamic period in terms of infection rates ranging from low to high incidence, as well as regarding the extent of measures to contain virus transmission that included two lockdown periods, on the one hand, and few restrictions and a massive influx of tourism in the summer of 2020, on the other.

## RESULTS

### Study cohort

**Fig. 1.**
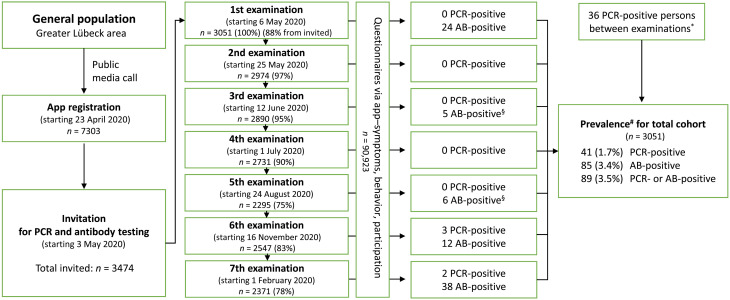
Study design and numbers of PCR- and anti-S1 IgG antibody (AB)–positive individuals. The ELISA cohort was prospectively followed from 6 May 2020 to 27 February 2021. *Two persons were PCR positive before the start of the study; §antibody data available from a subset with initially increased antibody titers only; #Kaplan-Meier estimator.

The Lübeck Longitudinal Investigation of SARS-CoV-2 Infection (ELISA) cohort (*n* = 3051) comprises 56% females and 44% males; the mean age is 45.6 years (SD, 15.2; range, 18 to 79 years) and represents ~1% of the local population in our catchment area. Participation rates were 75 to 98% across all seven time points (Fig. 1). Demographic details of the cohort and its two overlapping subgroups (the high-exposure subgroup includes 523 persons from the population-matched subgroup) and results of the SARS-CoV-2 PCR and antibody testing are summarized in Table 1.

**Table 1.** Study participants and group-wise results of antibody screening. Note that there is overlap of the population-matched and the high-exposure subcohorts with 523 persons from the population-matched group also having high exposure. AB, antibody.

		**Value**		**AB- or PCR-positive prevalence**		
		***N***	**(%)**	***N***	**%**	**(CI)**
**ELISA cohort (total)**		3051	(100)	89	3.5	(2.8–4.2)
**Gender**	Women	1710	(56)	45	3.1	(2.2–4.0)
	Men	1341	(44)	44	3.9	(2.8–5.0)
**Age**	18–39	1199	(39)	35	3.7	(2.5–5.0)
	40–59	1164	(38)	31	3.0	(2.0–4.1)
	60–79	688	(23)	23	3.6	(2.2–5.1)
**Flagged as high-contact person**		1645	(54)	47	3.6	(2.6–4.6)
**Education**	Low	916	(30)	33	4.4	(2.9–5.9)
	High	2127	(70)	55	3.0	(2.2–3.8)
**Population-matched subcohort**		1929	(100)	57	3.4	(2.6–4.3)
**Gender**	Women	1046	(54)	17	2.3	(1.1–3.5)
	Men	883	(46)	40	4.4	(3.1–5.8)
**Age**	18–39	669	(35)	18	3.5	(1.9–5.2)
	40–59	656	(34)	16	2.7	(1.4–4.0)
	60–79	604	(31)	23	4.1	(2.5–5.8)
**Flagged as high contact person**		523	(27)	15	3.8	(1.9–5.8)
**Education**	Low	550	(29)	21	4.5	(2.6–6.4)
	High	1375	(71)	36	3.0	(2.0–4.0)
**High-exposure subcohort**		1645	(100)	47	3.6	(2.6–4.6)
**Police, army, and firemen**		90	(5)	4	4.5	(0.2–8.9)
**Nurses**		236	(14)	13	12.2	(5.0–19.0)
**Other medical staff**		41	(2)	2	4.7	(0.0–10.9)
**Sales and distribution**		186	(11)	5	3.2	(0.4–6.0)
**Daycare teachers**		131	(8)	5	4.2	(0.6–7.9)
**School teachers**		160	(10)	2	1.5	(0.0–3.6)
**Students**		255	(16)	9	4.2	(1.5–7.0)
**Physician, outpatient sector**		122	(7)	1	0.9	(0.0–2.7)
**Physician, hospital**		142	(9)	3	2.7	(0.0–5.8)
**Other**		282	(17)	3	1.4	(0.0–3.0)

### Study setting

**Fig. 2.**
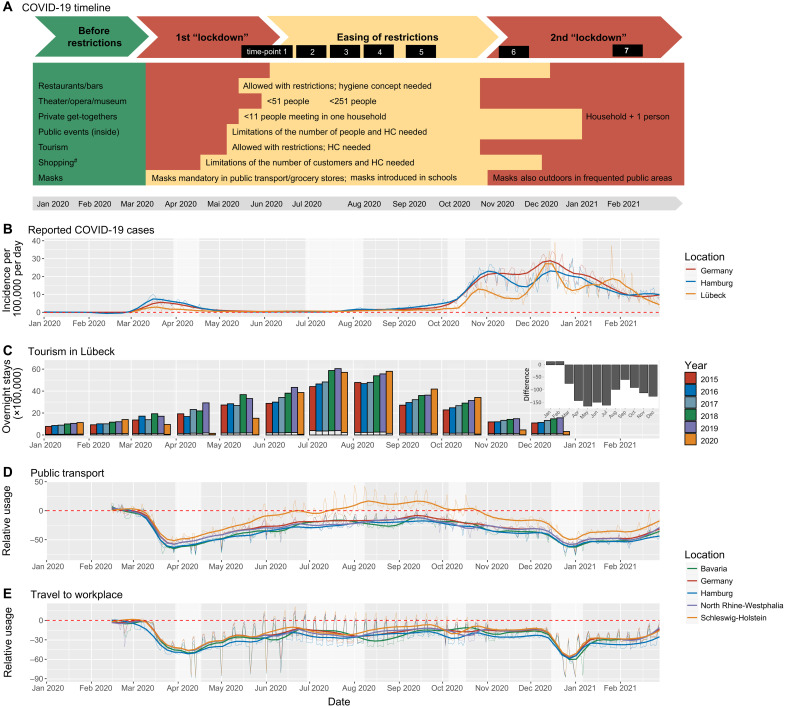
Study setting including lockdown measures, officially reported COVID-19 cases, tourism, public transport, and travel to workspace in the study area compared to other regions in Germany. (**A**) Lockdown measures in Lübeck, Schleswig-Holstein, Germany; green, no restrictions before the pandemic; yellow/orange, restriction/obligations; orange, more severe restrictions; red, forbidden/closed/most severe measures; the different time points of the ELISA study are shown in the black boxes; #except grocery stores, bakeries, and gas stations. HC, hygiene concept; detailed information about lockdown measures, and references are presented in table S1. (**B**) Reported SARS-CoV-2 infections in Lübeck, Hamburg, and Germany. (**C**) Overnight stays in the Lübeck area from the years 2015 to 2020. Black bars indicate the number of foreign tourists and the difference in overnight stays of foreign tourists in 2020 compared to 2015 to 2019 (inset). (**D** and **E**) Usage of public transportation and travel to work in Schleswig-Holstein, Germany, and three other German states. Data obtained from Google and reported as relative change from January/February 2020. The thick lines denote a LOESS fit to the daily data. The light gray backdrops indicate school holidays in Schleswig-Holstein.

To place our actual study results into context, we used publicly available data on infection rates, mobility, and tourism (Fig. 2). For this, data were obtained from Google COVID-19 Community Mobility Reports (www.google.com/covid19/mobility/, accessed 19 March 2021), from the Robert Koch Institute Covid-19 Datahub (https://npgeo-corona-npgeo-de.hub.arcgis.com) and by the tourism authority of the city of Lübeck. The greater Lübeck area is located on the Baltic Sea coast (fig. S1) and a tourist magnet with >2 million overnight stays in 2019 and an average of >50,000 visitors per day with high season in the summer. Until the second wave of the COVID-19 pandemic in Germany, Lübeck was a low-prevalence area (Fig. 2B). While there was practically no tourism during the two lockdowns in March and December, tourism in the summer and fall (June to October 2020) reached or even surpassed the already high influx of tourists in previous years (Fig. 2C). These numbers were supported by mobility data from Google showing a significant increase in the use of public transportation relative to all of Germany (*P* < 10 × 10^–30^, moderated *F* test) including other touristic hotspots (Fig. 2D). Travel to workplaces, however, remained similar across all states and Germany (*P* = 0.39, moderated *F* test) (Fig. 2E).

### Acute PCR-confirmed SARS-CoV-2 infections and anti-S1 IgG levels

**Fig. 3.**
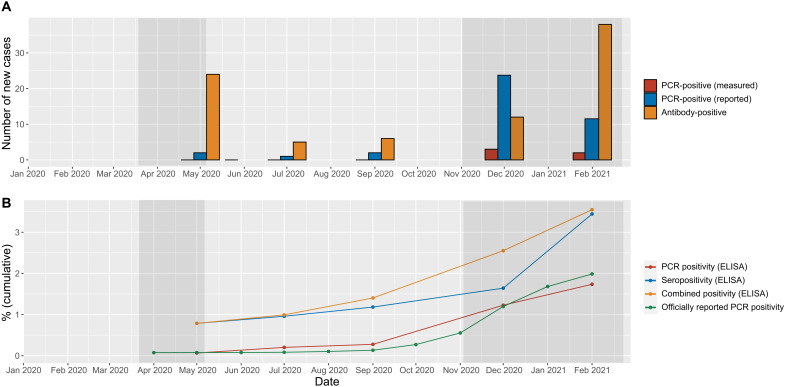
Longitudinally assessed PCR and seropositivity in the study cohort. SARS-CoV-2 virus and anti-S1 IgG profiles from May 2020 to February 2021. (**A**) Number of PCR-confirmed SARS-CoV-2 infections and individuals with anti-S1 IgG of >1.1 per time point. PCR-confirmed infections are shown in blue (reported in the study app) and red (positive PCR test at the study center), respectively. Newly detected anti-S1 IgG-positive cases are depicted in orange. (**B**) Frequency of PCR-confirmed infections within the study compared to officially reported results and anti-S1 IgG positivity rates. Data are presented in a cumulative fashion, whereby study participants were classified as positive at all future time points starting with their first positive test. Officially reported frequency of positive PCR results is shown in green (https://npgeo-corona-npgeo-de.hub.arcgis.com; rates of the last day of the month in Lübeck are shown). PCR positivity (reported and tested) at the study center is depicted in red, the total frequency of positive antibody results within the study cohort in blue, and combined positivity (PCR-confirmed infections and positive anti-S1 antibodies) in orange. The gray bars on the right- and left-hand sides of each panel indicate lockdown periods.

Two study participants reported PCR-confirmed SARS-CoV-2 infection before the first examination. The number of individuals with reported PCR-confirmed SARS-CoV-2 infection in the study app increased over the course of the study and reached *n* = 40 in February 2021. In addition, three and two individuals were tested positive at the study center in November/December 2020 and February 2021, respectively. Twenty-four participants were seropositive at baseline; 61 developed anti-S1 IgG levels above the cutoff over the study course (Fig. 3A). In relative numbers, the seropositivity rate within the study cohort increased slightly until September 2020, was higher in November/December 2020, and was even more so in February 2021 (Fig. 3B). The frequency of PCR-confirmed SARS-CoV-2 infections showed a similar tendency, however, with a peak in November/December 2020 (Fig. 3B).

**Fig. 4.**
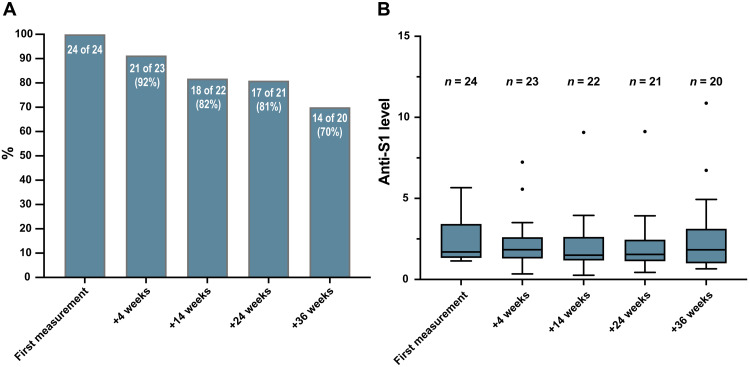
Antibody titer stability over the study course. (**A**) Of 24 individuals with anti-S1 IgG of >1.1 at baseline, the majority was available for follow-up examination. Individual antibody levels decreased below the cutoff in a proportion of individuals. The bars depict the percentage of participants still reaching anti-S1 IgG of >1.1 4, 14, 24, and 36 weeks after baseline measurement, respectively. (**B**) Medians of anti-S1 IgG levels of individuals considered antibody positive at the first measurement are shown at the following time points, notwithstanding if the individual antibody levels were still above the cutoff. Data are presented as box and whisker plots and the box extends from the 25th to the 75th percentile. The line in the middle of the box represents the median, and Tukey whiskers are used.

The stability of anti-S1 IgG antibody titers over time is depicted in Fig. 4. Within the 1-year observation period, 89 persons (3.5%) were antibody positive (*n* = 85) and/or had a positive PCR test result (*n* = 41) (Table 2). Prevalence of PCR or seropositivity in the population-matched subcohort was almost twofold but not significantly higher in men than in women {4.4% [95% confidence interval (CI), 3.1 to 5.8%] versus 2.3% (95% CI, 1.1 to 3.5%)}, and increased risk was detected in several high-exposure groups. The highest prevalence of 12.2% (95% CI, 5.0 to 19.0%) was observed for nurses, resulting in a 2.6-fold (95% CI, 1.78 to 4.99) increased risk of anti-S1 IgG antibody/PCR positivity for nurses compared to persons without occupational risk, followed by other medical staff, police, army, firemen, and students. Assuming that antibody-positive findings represent the true prevalence, about 92% of all infections were missed by PCR testing in May 2020 (*n* = 22 of 24). By February 2021, only 29% of the newly identified antibody-positive cases (11 of 38) remained undiagnosed (Table 2).

**Table 2.** PCR- and antibody-positive cases. The table shows absolute and relative numbers (*n*) for positive PCR and antibody measures and the combination thereof (PCR^+^ or AB^+^) for different time periods including the corresponding number of individuals at risk. For AB^+^ cases, we determined the numbers of cases that were “detected” by PCR testing before a positive antibody result was obtained. AB^+^ cases not detected by PCR were defined as “missed.” The ratio of “detected to missed” indicates how many AB^+^ cases were missed per PCR-detected case. For column “PCR^+^ or AB^+^,” an event was assigned to the time of the first occurrence of positive PCR or antibody test. Column “PCR^+^”: 39 of 41 PCR^+^ had antibody measured after a positive PCR test. Thirty-seven of them were AB^+^, and two were AB^−^ at the end of the study. The two remaining PCR^+^ cases had been vaccinated before antibody measurement.

	**PCR^+^ or AB^+^**		**PCR^+^**		**AB^+^**				
	At risk *n*	Events *n* (%)	At risk *n*	Events *n* (%)	At risk *n*	Events *n* (%)	Detected by PCR before AB^+^ *n* (%)	Missed by PCR *n* (%)	Ratio detected: missed
**May 2020**	3051	24 (0.79%)	3051	2 (0.07%)	3051	24 (0.79%)	2 (8.3%)	22 (91.7%)	1:11
**July 2020**	2894	5 (0.17%)	2923	1 (0.03%)	2894	5 (0.17%)	1 (20.0%)	4 (80.0%)	1:4
**September 2020**	2659	8 (0.30%)	2701	2 (0.07%)	2665	6 (0.23%)	0 (0.0%)	6 (100.0%)	-
**December 2020**	2580	29 (1.12%)	2613	24 (0.93%)	2581	12 (0.46%)	7 (58.3%)	5 (41.7%)	1:0.7
**February 2021**	2057	23 (1.12%)	2098	12 (0.57%)	2073	38 (1.83%)	27 (71.1%)	11 (28.9%)	1:0.4
**Total**		89 (3.46%)		41 (1.65%)		85 (3.44%)	37 (43.5%)	48 (56.5%)	1:1.3

Of 85 individuals with positive antibody results, 37 had had a positive PCR test. An additional 35 participants were antibody positive at at least one subsequent time point, leading to a confirmation of seropositivity in 85%. Of the remaining 13 participants with a single positive antibody test, 12 developed seropositivity late in the course of the study with no follow-up available.

### Analysis of risk stratification, behavior, quality of life, and correlation with antibody profiles

**Fig. 5.**
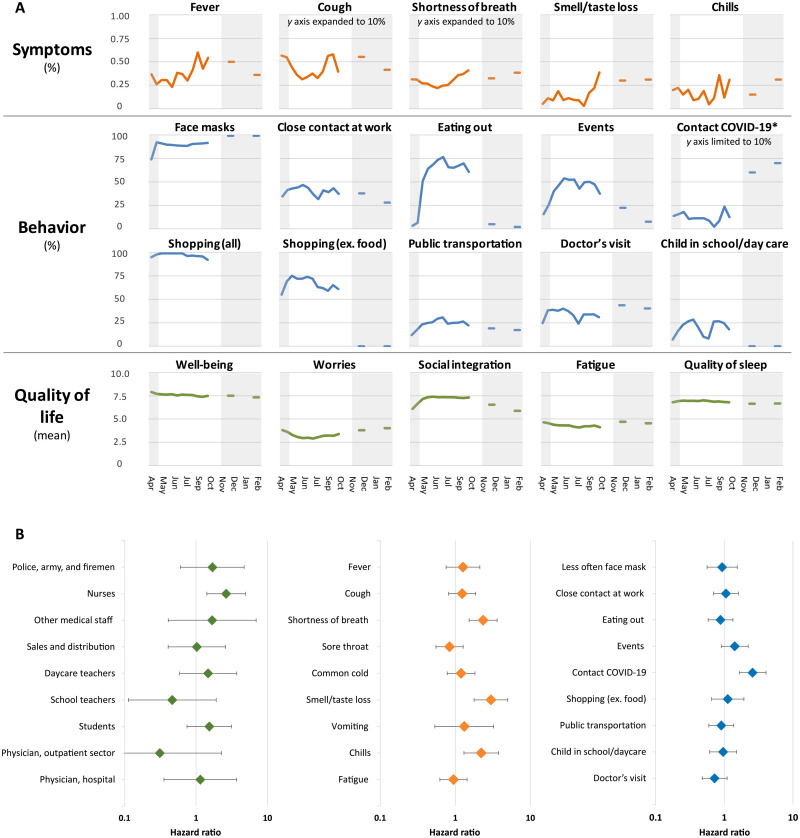
Symptoms, behavior, quality of life, and occupation over the study course and related to COVID-19 positivity. (**A**) Symptoms (orange), behavior (blue), and quality of life (green) during the pandemic from April 2020 to February 2021, aggregated by 2-week intervals on the individual person’s level. Symptoms and behavior are shown as percent values. The *y* axis is scaled to 1% for symptoms, 100% for behavior, and for some variables set to 10% as indicated; quality-of-life variables are shown as the mean value of a score ranging from 1 to 10 (1, lowest; 10, highest). The gray bars on the right- and left-hand sides of each panel indicate lockdown periods. At examination time points 6 and 7, questionnaires were no longer obtained every 3 days but only at two individual time points each. (**B**) Forest plot depicting hazard ratios (HRs) for antibody- or PCR-positive results for occupation (left), selected symptoms (middle), and behaviors (right).

The development of symptoms, behavior, and quality of life during the pandemic (Fig. 5A) is based on self-reports from 3051 study participants reported in 90,923 electronic questionnaires until February 2021. Frequencies of symptoms of infection rose between lockdown periods and dropped during the second lockdown. Behaviors were either stable (e.g., use of face masks) or increased in frequency between lockdowns in relation to the alleviation of the lockdown measures (e.g., eating out) and dropped again sharply during the second lockdown. The dip in frequency of many behaviors in the summer is related to the 6-week summer vacation period. Contact to patients with COVID-19 increased during the second lockdown. Quality of life remained stable for all included parameters, except for a moderate drop in social integration during the second lockdown period (Fig. 5A).

**Fig. 6.**
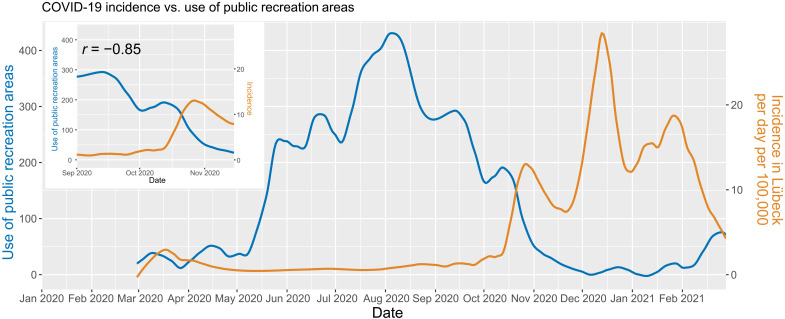
Reverse correlation between the use of public recreation areas (mobility data) and SARS-CoV-2 infections in the study area. The blue and orange lines depict the LOESS-smoothed “use of public recreation areas” in Schleswig-Holstein based on Google Mobility Data and the number of reported COVID-19 infections in Lübeck over the time course of March 2020 until March 2021. The top-left inset depicts the time from 1 September to 15 November during which the use of public parks declined greatly, and incidences rose in a highly anticorrelated manner (*r* = −0.85, Pearson correlation). The mobility data are based on the relative change relative to the 5-week period 3 January to 6 February 2020 and has been obtained from www.google.com/covid19/mobility/, accessed on 31 March 2021. LOESS smoothing is based on 10% of data points.

The occurrence of three of the self-reported symptoms was associated with anti-S1 IgG antibody/PCR positivity: loss of smell/taste [hazard ratio (HR), 2.98 (95% CI, 1.78 to 4.99)], shortness of breath [HR, 2.35 (95% CI, 1.53 to 3.61)], and chills [HR, 2.21 (95% CI, 1.30 to 3.76)] (Fig. 5B and table S2). Notably, the predictive value of these symptoms was low [e.g., only 18 of 244 (7.4%) persons with impairment of smell or taste were infected]. Analysis of different behaviors revealed “contact to persons with COVID-19” as the most relevant factor for testing positive [HR, 2.58 (95% CI, 1.65 to 4.04)] (Fig. 5B). Other factors, such as frequent use of public transportation, shopping, or close contacts at work, did not affect infection rates. Mobility data revealed an association between the decrease in the use of public recreation areas—likely indicating reduced outdoor activities after the summer—and the increase in SARS-CoV-2 infections in the study area (Fig. 6).

## DISCUSSION

The ELISA study is the first to provide longitudinal data throughout the SARS-CoV-2 pandemic on a prospectively followed, population-based cohort. Compared to the earlier point-of-care test approaches (*17*), it is further unique for its comprehensive design of seven rounds of PCR and antibody testing, combined with close digital tool–based recording of symptoms, behavior, and well-being. There is a critical need to identify specific groups and situations associated with an increased risk for SARS-CoV-2 infection (*18*), as such knowledge is urgently needed to inform choice, the timing of implementation (*16*), and duration of preventive measures. Currently, only modeling or cross-sectional studies with relatively small sample sizes or longitudinal studies covering a shorter time interval are available (*3*–*15*, *19*). Moreover, in most countries, including Germany, no structured population-based measures of high methodological quality have been implemented as yet, and data assessed by the health authorities still remain to be of insufficient quality. The high retention rate and acceptance among our study participants over a 1-year period confirm the feasibility of large-scale, long-term surveillance studies. As monitoring of 1% of the local population successfully mirrored reported infection rates of the region with acceptable precision, our study design may serve as an efficient and cost-effective model that is easily adaptable to different settings, regions, and stages of the pandemic.

Despite a large influx of tourism and mobility in the summer, as well as an unexpected rise of infection rates in the previously low-incidence study region in the fall/winter 2020/2021, overall antibody seropositivity remained low across all study time points and profoundly lower compared to high-prevalence areas such as Tirschenreuth (Germany) (*5*), Val Gardena (Italy) (*20*), or New York City (United States) (*21*). This further stresses the vulnerability of the vast majority of the population and the pivotal role of vaccination. Of particular note is the marked decrease in the proportion of undetected SARS-CoV-2 infections from 92% in May 2020 to 29% in February 2021, which is due to vastly increased testing efforts over the course of the pandemic. As a consequence, at the beginning of the pandemic, the true incidence may have been up to 11 times higher than the official figures, confirming early estimates (*22*).

Several COVID-19–linked symptoms (*23*), infection risk-associated behaviors, and higher occupational exposure in health care personnel (*24*), police, firemen, and students were confirmed or newly identified as plausible risk factors for SARS-CoV-2 infection. In contrast, other potential risk factors did not contribute to the risk of infection, such as use of public transportation (*13*), eating out (*25*), or having a child in school (*15*, *26*). Another important area of discussion relates to schools as a relevant driver of the pandemic. In our study, only 2 of 160 teachers were antibody positive after 1 year, confirming results from a prospective study conducted in primary schools in England (*27*). Other studies have used mobility networks to inform reopening (*28*) and identified selected ethnicities (*29*) and individuals with close household contacts (*30*, *31*) to be at higher risk of infection. Furthermore, the level of social mixing outside schools has been identified as a key factor influencing the effect of school closure (*32*).

Despite the massive influx of tourism from Germany into the Lübeck area in the summer of 2020 in parallel with an almost complete ease of lockdown restrictions and high mobility of the study population itself, we did not observe an increase in infection rates, suggesting that alleviating measures was safe. Notably, all of these findings have to be interpreted in the context of continuous hygiene measures and the overall low prevalence in our study area. Hence, the potential generalizability of these results to high-prevalence areas would need to be further explored. For example, some of the identified risk factors may be less relevant in regions of high prevalence because the overall infection risk overrides the risk conferred by specific factors, such as occupational exposure. With respect to the transferability of our study design, it would be scalable at the national level and could also be adapted to international settings. While easily accessible study centers like ours could likely be set up in most places, scarcely populated areas or specific population subgroups, such as refugees, may best be reached by mobile teams. However, despite limited interpretability due to the small size of individual subgroups and low predictive values in our present study, we do provide important data to inform the ongoing discussion of lockdown measures, as well as of prioritization of vaccination of specific at-risk groups in areas of limited access to vaccines.

The sharp rise in infection rates in late 2020 raises the important question as to whether we could have predicted the “second wave.” Expectedly, contact with infected individuals was the strongest sole risk factor for SARS-CoV-2 infection in our study. We hypothesize a fast rise in diffuse transmission, as soon as incidences surpass a critical threshold and quickly reach exponential growth when no (new) lockdown measures are promptly imposed, and the population is still nonvaccinated. While there were no changes of behavior or emerging differences in our high-exposure group heralding an imminent second wave, we noted a strong anticorrelation between the rise in the number of infections in Lübeck and the use of public recreation areas indicative of more indoor activities that may have fostered contact with infected individuals. Innovative ways of forecasting imminent rises in infection rates are an urgent and still largely unmet need. In this regard, a recent study evaluated digital data streams as early indicators of state-level COVID-19 activity in the United States and found increases in digital data stream activity to anticipate a rise of confirmed cases and deaths by 2 to 3 weeks (*33*).

There is a well-established need to correct for sensitivity and specificity of serological assays in cross-sectional antibody studies (*34*). However, we were able to confirm the vast majority of positive antibody results by either a positive PCR test and/or a second, independently assessed antibody test above the cutoff, highlighting the robustness of longitudinally obtained results.

Thus, the strengths of our study include its comprehensive and longitudinal design, the quality of virus and antibody testing, and exceptionally high cohort retention. Incentives for participation were threefold: In particular, at the beginning of the pandemic, testing for SARS-CoV-2 infection was not yet widely available and expensive. The probands were grateful for the free testing and perceived the (mostly) negative test results as reassuring. Second, at each of the seven time points, the participants were given a small box of candy featuring the ELISA study logo. Third, many probands expressed their gratitude for the opportunity to make a personal contribution to a better understanding and containment of the pandemic. Additional strengths of our study design are the dynamic nature of the study region in terms of tourism, mobility, and the sudden change from a low- to a high-prevalence region. Another unique feature is the combination of a wealth of digital health and detailed behavioral data. Besides the above-discussed constraints regarding generalizability, transferability, and interpretability of our findings, a further limitation might be the overrepresentation of highly motivated study participants with an above-average education level.

Although vaccination had only become available at our last examination time point (~10% of the study cohort was vaccinated in February 2021), we provide a model for effective surveillance that is transferable and adaptable to other settings and regions and that allows to also monitor reinfection rates and vaccination breakthroughs. These are important and timely considerations, especially given the expected further spread of the virus and of new variants.

According to current knowledge and newly introduced surveillance options, we would like to propose several additions to an updated protocol for future studies including, but not limited to, sequencing of virus variants, increasing the number of surveillance time points including antigen (self)testing, and implementing the use of dried blood spot filter cards to determine antibodies circumventing the need for venipuncture. At the present stage of the pandemic, one would also obtain information about time, number, and type of vaccinations, as well as about vaccination breakthroughs.

In conclusion, we (i) demonstrate that infection rates were vastly underestimated at the beginning of the pandemic, highlighting the necessity of intense testing; (ii) provide an easy-to-be-adapted model for effective, population-based surveillance of the current (and future) pandemic(s); and (iii) demonstrate that easing of lockdown measures appears safe even over several months at times of low prevalence rates, suggesting that a well-working surveillance system may serve as a feasible alternative to strict lockdown measures and as key for future protection against SARS-CoV-2, as well as for preparedness for future pandemics due to potential novel infectious agents.

## MATERIALS AND METHODS

### Study oversight and design

The ELISA study was approved by the ethics committee of the University of Lübeck (Az. 20-150). Participants were invited to take part in the study through announcements in the local press, posters, and leaflets and by advertisement on the official homepage of the city of Lübeck by its mayor. Detailed study information was provided on ELISA study’s website (https://elisa-luebeck.de), by telephone counseling, and in newsletters. Within 2 weeks in April 2020, 7303 adult inhabitants of the greater Lübeck area (approximately 300,000 inhabitants) downloaded the dedicated ELISA study app. All 7303 participants were invited to fill out the study questionnaire, covering retrospectively also the period from March 2020; 3474 were selected for subsequent SARS-CoV-2 testing. Of these, 2145 individuals matched by age and sex distribution of the study region were randomly drawn to comprise a population-based group. An additional 1329 individuals were selected to enrich a potential high-exposure group based on profession requiring intense and/or frequent contact with other people, such as health care personnel (Fig. 1). A total of 3051 persons (88%) came to the study center for initial screening (ELISA cohort). All cohort members were invited to the study center seven times between early May 2020 and February 2021, spanning the end of the first lockdown, a massive influx of tourism in the summer, the steep rise of infection rates in the fall/winter 2020/2021, and the second lockdown (Fig. 2A). All participants were tested for current or previous SARS-CoV-2 infection using nucleic acid and anti-S1 (extracellular S1 part) IgG antibody testing with the support of a mobile app–based questionnaire, electronic scheduling, and result reporting system. The overall study costs per participant amounted to ~600 USD for the seven time points combined.

### Detection of SARS-CoV-2 infections

Trained medical students performed deep nasal and oropharyngeal swabs (*35*) under the supervision of medical doctors and combined them into a single tube for each participant before RNA isolation. SARS-CoV-2 quantitative real-time PCR (RT-PCR) from dry swabs was performed at the National Reference Center for Mycobacteria at the Research Center Borstel, Leibniz Lung Center (*36*), and the University Hospital Schleswig-Holstein, Lübeck, using validated PCR platforms. External supporting sites included the LADR Central Lab Dr. Kramer & Colleagues, Geesthacht and Centogene GmbH, Rostock (*37*), using accredited SARS-CoV-2 diagnostic pipelines. Briefly, dry swabs were suspended within 24 hours and processed for nucleic acid extraction using QIAamp Viral RNA Kits (QIAGEN, Hilden) or the NucliSens easyMAG platform (Biomérieux), according to the manufacturers’ instructions. Nucleic acids were eluted, and quantitative RT-PCR was performed according to the manufacturers’ instructions using the first World Health Organization Emergency Use (*38*) listed in vitro diagnostics coronavirus (COVID-19) genesig RT-PCR assay (Primerdesign Ltd.), or the iTaq Universal Probes 1-Step kit (Bio-Rad, Feldkirchen) in conjunction with primers and probes from the SARS-CoV-2 (2019-nCoV) CDC qPCR Probe Assay (Integrated DNA Technologies, Leuven, Belgium). All in-house PCR assays were validated and passed the interlaboratory quality control (INSTAND, Düsseldorf).

For antibody testing, a venous blood sample was drawn. The EUROIMMUN SARS-CoV-2 S1 IgG (#EI 2606-9601-2 G) enzyme-linked immunosorbent assay was performed according to the manufacturer’s instructions for examination time points 1, 6, and 7 and partially for time points 3 and 5 for participants with increased anti-S1 IgG antibody titers at time point 1. Antibody titers against the S1 antigen of >1.1 were considered positive. Values between 0.8 and 1.1, which are considered borderline, were not included in further analyses (*39*). All persons with antibody-positive results were contacted by telephone to inquire about potential external PCR-positive test results obtained in between study visits.

### App data

Participants were initially asked to answer a baseline questionnaire including demographic data, level of education, symptoms of infection and allergies, profession, travel, activities of daily living, details of adherence to lockdown and later precautionary measures, contact with children, and self-reported results of previous SARS-CoV-2 tests (see Questionnaires in the Supplementary Materials). Participants were asked to repeat this app-based questionnaire [using an adaptation of the MillionFriends App by Perfood GmbH (https://perfood.de/)] every 3 days until September 2020, corresponding to time points 1 to 5. In December 2020 and February 2021, respectively, participants were asked to fill out the questionnaire only once (time points 6 and 7).

### Statistical analysis

Data of the ELISA study were stored in a central study database and pseudonymized for subsequent analyses. We used frequency tables and cross tabulations to characterize the ELISA cohort and its subgroups. The prevalence of PCR- or antibody-positive participants was estimated using the Kaplan-Meier method. Persons who did not attend all study visits were censored after their last visit. We also censored all vaccinated participants at the last time point (*n* = 269, 8.8% of the cohort). To explore the potential impact of symptoms on PCR- or antibody-positive results, we marked a person as symptom positive if a symptom was present for at least one time point during the study period. For analyzing behavior and quality of life, we calculated a mean behavior score over time for each variable and participant and dichotomized the variables at the median into low/high levels of behavior. We used Cox regression to estimate HRs for symptom and behavior variables and calculated 95% CIs for prevalence and risk estimates. Differences in temporal trends were analyzed by a moderated *F* test on a regression spline curve with 5 degrees of freedom.

To analyze the detection rate of COVID-19 cases by PCR testing, the absolute and relative numbers for positive PCR and antibody measures and the combination thereof were assessed including the corresponding number of individuals at risk at different time points. We determined the number of antibody-positive cases that were detected by PCR testing, i.e., the positive antibody result was obtained after that of a positive PCR test. Next, a ratio of antibody-positive cases with a previously positive PCR test (“detected cases”) to antibody-positive cases without a previously positive PCR test (“missed cases”) was calculated, indicating how many antibody-positive cases had escaped PCR testing at previous testing time points.

## Supplementary Materials

### This PDF file includes:

Fig. S1 Tables S1 and S2 Questionnaires

## Notes

View/request a protocol for this paper from *Bio-protocol*.
